# Curiosities of French Medical Literature

**Published:** 1860-03

**Authors:** 


CURIOSITIES OF FRENCH MEDICAL LITERATURE.—Under this caption a critic in a late number of the London *Medical Times and Gazette*, asks the question,—

"Why cannot a Frenchman copy an Englishman's name correctly? There is something really strange in the fact: that our French friends insist now, as they have always insisted, in rarely giving us our true cognomens. Can any of your readers give an explanation of this psychological obliquity? We rarely ever find an Englishman miswrite the name of a Frenchman, and for this reason we think the queer figures French authors make us cut hardly fair. A page of a journal—*L'Union Médicale*—lies before me, from which I learn that M. Léfort, Aide d'Anatomie of the Faculty of Medicine of Paris, places upon the Bureau of the Society of Chirurgery of Paris, a voluminous memoir on resection of the knee. He contents himself, however, with reading a *résumé* of the same—a piece of modesty which, by the way, might occasionally be usefully practiced in some of our own learned societies. Running my eye down this page of *résumé*, I find the names of my blessed departed countrymen thus handled: *William* Wright took off five and a half inches (in 1738) from the femur of a boy called Herd Ramsden. In 1739 Benjamin Gooch, assistant to Dr. *Amyas*, of Norwich, did something of a like kind. In 1760 *Waisman de Shispton* (*Angleterre*) cut away some of a humerus. In 1762 *Pelkin*, Surgeon of *Nortorich en Cheslire*, first commenced resecting manœuvres on the knee; and then Bent, of Newcastle; and *Owed*, in 1778. renewed attempts on the shoulder. *Owed*, however, in 1773, was the first honorable resector of the wrist. And in 1782 Moreau did a great thing without knowing it had already been done in England, three years before the translation of *Parck's* memoir appeared in France. Lastly turns up M. Jones de *Gersey*. A little further on I hesitate, for I may be in error, if I surmise that M. *Euchsen* means Mr. Erichsen. I think, sir, you will agree with me that there is something queer in the typographical bumps of men, who slaughter our countrymen's names in such a fashion."

Frenchmen, particularly medical Frenchmen, are proverbially miserable English scholars, and hence it is not surprising that they should be stupidly ignorant of everything relative to English and American medicine and surgery. Almost the only modern Frenchman who forms an exception to this rule is Velpeau, whose Treatise on Operative Surgery displays great familiarity with everything that has been achieved in this particular department in this country and Great Britain. In 1805, a Frenchman, J. B. La Roche, alluding to the seton, as proposed by Dr. Philip Syng Physick, for the treatment of ununited fracture, supposing the family name of the father of American surgery to be that of his profession, ascribed the honor of the invention to Monsieur Philip S., physician. That such an error should have been committed early in this present century, is not at all surprising, especially when we remember the peculiarity of Physick's name; but that such mistakes in orthography as those pointed out in our foreign contemporary should occur in 1859, at a time when there is so much intercourse between France and England, is, to say the least, not only extremely ludicrous, but very unpardonable. Frenchmen are, however, wonderfully conceited, and their creed evidently has long been that there is but one God and one France.

